# 1426. Empiric Antimicrobial Prescribing for Urinary Tract Infections in Patients Discharged from the Emergency Department

**DOI:** 10.1093/ofid/ofab466.1618

**Published:** 2021-12-04

**Authors:** Megan A Rech, Brett Faine, Priyanka Vakkalanka, David A Talan

**Affiliations:** 1 Loyola University Medical Center, Maywood, Illinois; 2 University of Iowa, Iowa City, Iowa; 3 Olive View-UCLA Medical Center, Sylmar, CA

## Abstract

**Background:**

Urinary tract infections (UTIs) are commonly treated infections in the emergency department (ED), accounting for 3 million visits annually and 15% of outpatient antibiotic prescriptions. The purpose of this study was to characterize empiric and definitive antimicrobial therapy for treatment of UTIs in a nationally representative sample of ED patients.

**Methods:**

This was a multicenter, retrospective cohort study utilizing the Emergency Medicine PHARMacy Research NETwork (EMPHARM-NET), a network of 15 geographically diverse EDs. Patients ≥18 years presenting to and discharged home from the ED with primary diagnosis code of cystitis, pyelonephritis, or UTI from 2018-2020 were included. We describe empiric intravenous (IV) and oral antibiotics used for the treatment of UTI in patients seen and discharged from the ED.

**Results:**

Of the 3779 ED patients treated for UTI, most were discharged from the ED (n=2483, 66%). Most patients were female (76.3%) and common comorbidities were hypertension (47.8%) and diabetes (26.5%). Most patients had uncomplicated (39.4%) or complicated (40.9%) cystitis. 1134 (45.6%) had a positive urine culture, most commonly *E. coli* (63%) and *K. pneumoniae* (13%). The most common antibiotics administered in the ED were ceftriaxone (19.7%), nitrofurantoin (6.2%), cephalexin (5.8%), and sulfamethoxazole/trimethoprim (SMX/TMP, 4.8%). The most common antibiotics prescribed at discharge where cephalexin (33.9%), nitrofurantoin (20.6%), SMX/TMP (12%), ciprofloxacin (8.2%), and cefdinir (8%). The mean length of treatment was 7.1 days (standard deviation 2.5 days). Overall, 454 patients returned to the ED within 30 days. The odds of returning to the ED within 30 days was higher in those that did not have appropriate empiric antibiotics based on susceptibilities (OR 1.37, 95% confidence interval 1.06, 1.78).

**Conclusion:**

This multicenter, retrospective cohort study describes ED patients discharged from the ED after UTI diagnosis. Patients presented most commonly for cystitis. Nearly half of discharged patients were culture positive. Antimicrobial selection varied; IV ceftriaxone and oral cephalexin were most commonly empirically utilized to treated patients with UTI. Inappropriate antimicrobial selection increased odds of a return ED visit within 30 days.

**Disclosures:**

**Megan A. Rech, PharmD, MS, BCCCP, FCCM**, **Spero** (Research Grant or Support) **Brett Faine, PharmD**, **Spero Therapeutics** (Research Grant or Support) **David A. Talan, MD**, **AbbVie** (Consultant)**GSK** (Consultant)**SPERO Therapeutics** (Grant/Research Support)

